# Frontostriatal Cognitive Staging in Parkinson's Disease

**DOI:** 10.1155/2012/561046

**Published:** 2011-12-06

**Authors:** Raúl de la Fuente-Fernández

**Affiliations:** Section of Neurology, Hospital A. Marcide, 15405 Ferrol, Spain

## Abstract

Cognitive impairment and behavioural disorders are often encountered in subjects with Parkinson's disease (PD). A simple PD-related frontostriatal cognitive dysfunction (PDFCD) staging is proposed. Executive dysfunction and mental fatigue (stage I), depression/anxiety (stage IIa), apathy/pain (stage IIb), and dementia (stage III) reflect a sequential process of dopamine depletion occurring in different regions of the striatum (stages I and II) and the frontal cortex (stage III). In addition to these nonmotor manifestations present in the unmedicated (OFF) state, the PDFCD model also predicts a number of complications related to dopaminergic treatment (ON state), from impulse control disorders (stages I and IIa) to hallucinations (stage IIb) and psychosis (stage III). Although the model admittedly needs further refinements, it provides a framework for hypothesis testing and may help clinicians optimize therapeutic strategies.

## 1. Introduction

Parkinson's disease (PD) is biochemically characterized by dopamine depletion [1, 2]. Although the loss of dopamine is particularly severe in the putamen, which explains the motor manifestations of the disease, other dopaminergic projections are also affected and contribute to the development of cognitive impairment and neuropsychiatric disorders [3, 4]. Thus, some degree of executive dysfunction is a virtually constant finding in PD, even in the early stages of the disease [5]. Apathy, depression, anxiety, and fatigue are present in one third of patients [6], and pain is also common [7]. Similarly, it has been estimated that approximately one third of PD subjects end up developing dementia [3, 4]. In this review, I will use a simple model to correlate these non-motor manifestations of the disease with different stages of frontostriatal dysfunction caused by dopamine depletion sequentially occurring in different regions of the striatum and the frontal cortex [8–11].

**Figure 1:**
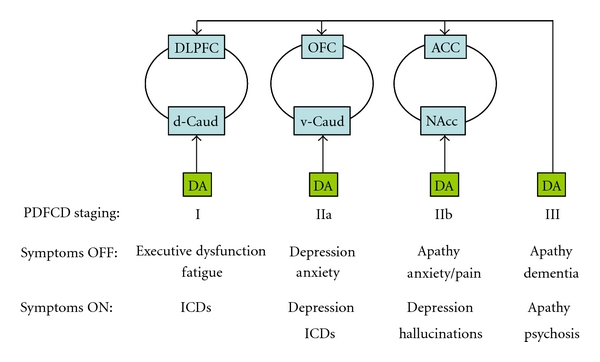
Parkinson's disease-related frontostriatal cognitive dysfunction (PDFCD) staging. Three major frontostriatal loops are shown: (1) the loop connecting the dorsolateral prefrontal cortex (DLPFC) with the dorsal caudate nucleus (d-Caud), (2) the loop connecting the orbitofrontal cortex (OFC) with the ventral caudate nucleus (v-Caud), and (3) the loop connecting the anterior cingulate cortex (ACC) with the nucleus accumbens (NAcc). Dopamine (DA) projections for these loops, as well as the direct dopaminergic projection to the frontal cortex, are schematically shown. Major symptoms, OFF and ON dopaminergic treatment, are detailed. ICDs: impulse control disorders.

**Figure 2:**
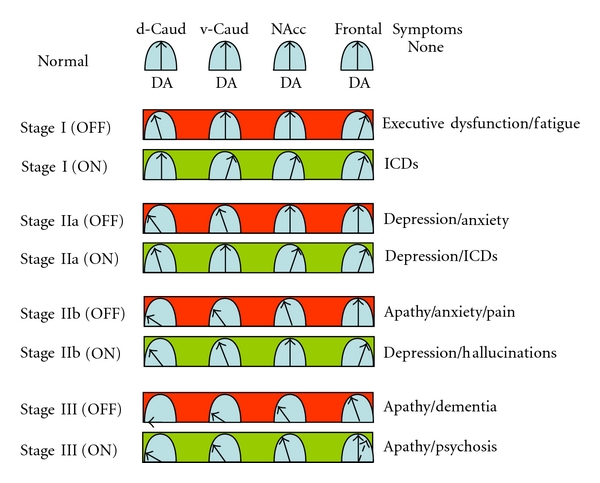
Parkinson's disease related frontostriatal cognitive dysfunction (PDFCD) staging with region-specific dopamine (DA) levels. Dopamine-related frontostriatal functioning is assumed to follow an inverted U-shaped dose-response curve. Predicted stage-specific dopaminergic function, both off and on dopaminergic treatment, is shown for dorsal caudate (d-Caud), ventral caudate (v-Caud), nucleus accumbens (NAcc) and frontal cortex. In the ON state, the inverted U-shaped curves represent DA levels if the patient is on levodopa, and DA tone if the patient is on a dopamine agonist. The direct dopaminergic projection to the frontal cortex seems to be initially upregulated, but with limited capability to increase further the dopaminergic tone in response to dopaminergic treatment (see text for details). ICDs = impulse control disorders.

The PD-related frontostriatal cognitive dysfunction (PDFCD) staging here proposed (Figure 1) assumes that dopamine-dependent frontostriatal functioning follows an inverted U-shaped dose-response curve (Figure 2). The PDFCD model is mostly based on neuroimaging data and clinical observations, and offers stage-specific clinical predictions off and on dopaminergic medication.

## 2. Dopamine Depletion and Dysfunction of Frontostriatal Loops

PD is characterized by a gradient of dopamine depletion in the striatum, with the putamen being the most affected region, followed by the dorsal caudate, and then the ventral striatum (ventral caudate and nucleus accumbens) [1, 2]. A combination of PD-specific and aging-related dopamine depletion [12, 13] determines the degree of cognitive/behavioural dysfunction.

Three major anatomical and functional frontostriatal loops are proposed to be sequentially affected in PD: first, the motor loop, which connects the supplementary motor area with the putamen; second, the cognitive loop, which connects the dorsolateral prefrontal cortex (DLPFC) with the dorsal caudate nucleus; and third, a “complex” limbic loop, with connections between the orbitofrontal cortex (OFC) and the ventral caudate nucleus, and between the anterior cingulate cortex (ACC) and the nucleus accumbens. These three functional frontostriatal loops have been well characterized both theoretically and experimentally [8–11]. In more advanced PD, the direct dopaminergic projection to the frontal cortex becomes also affected, leading to cortical dopamine depletion [14, 15]. There is evidence that the release of dopamine, at both the striatal and cortical level, facilitates loop functioning [8–11]. Alterations in the motor loop are crucial because they serve to make the clinical diagnosis, signalling that PD pathology has reached midbrain dopamine cells [16]. However, the PDFCD model only applies to the cognitive/limbic loops and the direct dopaminergic projection to the frontal cortex.

In the early stages of PD, frontal lobe dysfunction is assumed to reflect cortical “deafferentation” in relation to striatal dopamine depletion. At later stages, cortical dopamine depletion likely contributes to frontal lobe impairment. In keeping with this notion, dementia in PD is related to the loss of dopamine cells in the medial part of the substantia nigra pars compacta [17] and ventral tegmental area [18], regions that originate direct dopaminergic projections to the cortex. Interindividual variability in the time course and degree of dopamine depletion in different striatal and frontal regions may explain the wide range of clinical manifestations encountered in PD. Although the PDFCD model is only based on striatal and frontal dopamine depletion, cortical Lewy body pathology, which typically occurs in advanced PD [16], will eventually contribute to the development of frank dementia [16, 19].

## 3. PDFCD Stage I: The Frontostriatal Cognitive Loop

### 3.1. Functional Analysis

The frontostriatal cognitive loop (DLPFC—dorsal caudate nucleus) is involved in executive function [5]. Normal performance of typical cognitive frontal lobe tasks, such as the Wisconsin Card Sorting Task or the Tower of London planning task, depends upon several frontal executive functions including working memory, attention, planning, and cognitive flexibility, all of them pertaining to this frontostriatal loop [20–23]. PD subjects off medication have impaired task set-shifting [24, 25], reduced basal ganglia activation during performance of the Tower of London test [26, 27], and show correlations between the degree of impairment on executive tasks and the degree of dopaminergic hypofunction in the caudate nucleus [28, 29]. These alterations are virtually constant in PD and suggest frontal lobe “deafferentation” caused by dopamine depletion in the dorsal caudate nucleus [24, 30]. There is some indication that such a “deafferentation” predicts incident dementia [31]. Interestingly, the direct dopaminergic projection to the prefrontal cortex seems to be hyperactive early in the course of the disease [32, 33], presumably as a compensatory mechanism (Figure 2). Nonetheless, dorsal caudate-dependent tasks are consistently associated with DLPFC hypoactivation [34].

### 3.2. Clinical Correlates

#### 3.2.1. OFF Dopaminergic Treatment

Clinical manifestations of frontal lobe dysfunction, including poor planning, defects in set-shifting, impaired working memory, and executive dysfunction, are common in PD without dementia [35] and correlate with a PD-related cognitive pattern of altered glucose metabolism [36]. This pattern is characterized by metabolic reductions in frontal areas, and relative metabolic increases, presumably compensatory, in the cerebellum. Fatigue (mental fatigue) can be present in PDFCD stage I (Figures 1 and 2) and may even precede the onset of motor symptoms [6].

#### 3.2.2. ON Dopaminergic Treatment

Dopaminergic treatment induces impulse control disorders (ICDs) in a number of PD subjects as the result of the overactivation of the “complex” frontostriatal limbic loop [37]. Hence, ICDs occur in PDFCD stages I and IIa (Figure 2; see next section for further details). In contrast to the dopaminergic projections to the striatum, the activity of the dopaminergic projection to the frontal cortex is not expected to be substantially altered by dopaminergic treatment, especially when it is overactive, because it lacks dopamine transporter sites and dopamine D2 autoreceptors [38]. In fact, the action of dopamine in the frontal cortex is mostly mediated by dopamine D1 receptors [39]. This might explain some of the clinical differences between levodopa therapy and treatment with direct dopamine agonists. While levodopa-derived dopamine stimulates D1 and D2 receptors, dopamine agonists predominantly stimulate D2 receptors.

## 4. PDFCD Stage II: The “Complex” Frontostriatal Limbic Loop

### 4.1. Functional Analysis

In PD, the damage to the dopaminergic projection to the ventral striatum (ventral caudate and nucleus accumbens) is less prominent than the damage to the dopaminergic projection to the dorsal striatum (putamen and dorsal caudate) [1, 12]. Still, ventral regions of the striatum also undergo substantial dopamine depletion (~60% dopamine loss) [1, 13].

Reversal learning tasks, which basically test for balance between “go” and “no-go” signals [40], are used to assess the “complex” frontostriatal limbic loop (OFC/ACC—ventral caudate/nucleus accumbens) [41]. As one would predict according to the regional differences in the degree of striatal dopamine depletion, PD subjects off medication perform much better in reversal learning tasks than in tasks involving the dorsal caudate circuitry [10, 41–43]. Conversely, dopaminergic therapy improves dorsal caudate related tasks and worsens reversal learning tasks [10, 41, 42]. This paradoxical effect of medication is probably due to the “over-dose” of a relatively normal ventral striatum [10, 42–45]. PD subjects tend to avoid negative outcomes when being off medication, and they are sensitive to positive outcomes when being on medication [40]. In other words, dopaminergic therapy favours “go” signals over “no-go” signals.

### 4.2. Clinical Correlates

Although it is admittedly difficult to functionally separate the two components of the “complex” frontostriatal limbic loop, there is some suggestion that the OFC—ventral caudate circuit modulates social/emotional behaviour and the ACC—nucleus accumbens circuit mediates motivation and integrates cognitive and emotional iterative networks [9]. Consequently, hypoactivation of the limbic loop, specifically the ACC—nucleus accumbens circuit, is expected to lead to apathy. Limbic loop hyperactivation, on the other hand, is expected to lead to impulsive behaviours.

#### 4.2.1. OFF Dopaminergic Treatment

Depression, anxiety, and apathy are common in PD [6, 46, 47]. Apathy still needs a clear definition. Most authors would agree that it refers to a lack of motivation [48]. In any case, it is increasingly recognized that apathy is not depression, although both disorders share a number of clinical characteristics, including psychomotor retardation, diminished interest, anergy, and lack of insight [49]. The proof of concept for a distinction between apathy and depression came from the observation that non-PD-depressed patients treated with selective serotonin reuptake inhibitors (SSRIs) sometimes develop apathy [50, 51].

Some imaging studies suggest that depression in PD is associated with dopamine depletion in the ventral striatum [52] and hypoactivation of the cingulate cortex [53, 54]. However, these results may need to be reassessed in view of the increasing awareness of a distinction between depression and apathy. Recent estimates suggest that apathy is more prevalent than depression in PD [6, 47], suggesting that many patients who are assumed to have depression could have apathy instead. In non-PD patients with depression, the ACC (specifically, its subgenual portion) has been found to be hyperactive, not hypoactive [55–57]. In addition, pathological and neuroimaging observations in Alzheimer's disease, where apathy is a very common phenomenon [47], indicate that there is a correlation between apathy and neurofibrillary tangles burden in the anterior cingulate cortex [58–60]. Likewise, apathy in PD correlates with reduced gray matter in the cingulate cortex [61]. Taken together, these observations suggest that depression corresponds to PDFCD stage IIa (hypofunction of the OFC—ventral caudate circuit), and apathy corresponds to PDFCD stage IIb (hypofunction of the ACC—nucleus accumbens circuit) (Figures 1 and 2). Naturally, PDFCD stage IIb also includes hypofunction of the OFC—ventral caudate circuit, which explains why depression and apathy occur sequentially and share several clinical characteristics.

Depression is sometimes a premotor manifestation of PD but more often appears after motor symptom onset [6, 46]. The underlying neurochemical bases may differ in both scenarios. Depression occurring during the pre-motor phase of the disease probably reflects Lewy pathology in raphe nuclei and locus coeruleus, originating serotonergic and noradrenergic abnormalities [16]. Accordingly, depression preceding PD motor symptoms should respond to conventional antidepressants, including SSRIs. Nonetheless, recent neuroimaging studies have challenged this concept by showing preserved serotonin transporter binding in the novo PD subjects [62]. The neurochemical basis of pre-motor depression remains, therefore, unclear; dopamine dysfunction could play a role. Depression occurring at later stages, on the other hand, is likely related to dopamine depletion in the ventral caudate (PDFCD stage IIa; Figure 2). In this case, treatment is problematic. Many antidepressants, particularly SSRIs, have failed to demonstrate efficacy in PD [63, 64]. In fact, SSRIs can paradoxically precipitate apathy. Neuroimaging studies provide some clues to explain this phenomenon. In non-PD subjects with major depression, antidepressants correct ACC overactivity and there is a correlation between the degree of relative ACC overactivity at baseline and the response to antidepressants [55]. In PD, treatment with SSRIs can lead to apathy by decreasing the activity of the ACC—nucleus accumbens circuit (i.e., SSRI-related functional transition from stage IIa to stage IIb).

Anxiety typically occurs during the motor phase of PD [6, 46]. Although other neurotransmitters are in all likelihood responsible for anxiety during the pre-motor phase of the disease, dopamine depletion in the ventral striatum seems to play a major role once the motor symptoms are established. Thus, the severity of anxiety is inversely correlated with dopamine/noradrenaline transporter binding in caudate, ventral striatum, and amygdala [52]. Animal experiments indicate that stress is associated with decreased dopamine release in the nucleus accumbens and increased dopamine release in the medial prefrontal cortex [65]. This observation suggests that anxiety occurs when the ACC—nucleus accumbens circuit is hypoactive and the direct dopaminergic projection to the frontal cortex is still functionally preserved (PDFCD stage IIb; Figure 2). However, PD subjects on dopaminergic therapy often present with a combination of depression and anxiety during the OFF periods, suggesting a connection between anxiety and PDFCD stage IIa. PD-related functional or pathological alterations in the amygdala [66], a limbic structure known to play an important role in anxiety disorders [67], could ultimately determine whether anxiety occurs in PDFCD stage IIa or stage IIb.

Apathy in PD is proposed to be associated with hypofunction of the ACC—nucleus accumbens circuit (PDFCD stage IIb; Figure 2). A recent study reported apathy in 23% of drug-naïve PD subjects [68], indicating that the limbic loop can be dysfunctional early in the course of the disease. At later stages, when PD subjects develop a combination of striatal and frontal dopamine depletion (PDFCD stage III), apathy is expected to become more prevalent and severe, with even further progression in more advanced PD, with the development of frontal Lewy body pathology [16, 19]. In this context, subjects with frontotemporal dementia have the highest prevalence rates of apathy across disorders with frontal dysfunction [47].

Pain is a nonmotor manifestation of PD known to be influenced by dopaminergic treatment [7, 69]. The PDFCD model suggests that pain (central pain) is related to dopamine depletion in the ventral striatum, specifically in the nucleus accumbens (PDFCD stage IIb; Figure 2). In support of this view, there is experimental evidence suggesting that the nucleus accumbens is involved in a dopamine-opioid network that modulates pain transmission [70, 71]. Pain is sometimes directly related to poor motor performance (dystonic pain) [7], reflecting dopamine depletion in the putamen.

It has already been mentioned that fatigue (mental fatigue) can be an early symptom of PD (PDFCD stage I) [6, 72]. More often, however, it is associated with depression and reduced motivation [6, 73], signalling PDFCD stages IIa and IIb, and possibly stage III as well. Serotonergic dysfunction may contribute to fatigue [74].

#### 4.2.2. ON Dopaminergic Treatment

Remarkably, the PDFCD model suggests that depression can be present during ON periods in stages IIa and IIb (Figure 2), explaining a relatively common clinical observation (i.e., PD subjects who are depressed off and on medication).

Clinical studies suggest that some 10% of PD subjects treated with dopaminergic medications develop ICDs, including pathological gambling, compulsive shopping, compulsive eating, hypersexuality, and even addictive behaviours [75, 76]. The PDFCD model suggests that ICDs are due to treatment-related hyperactivations of the ACC—nucleus accumbens circuit, and should therefore occur in stages I and IIa (Figure 2). Experimental observations support this notion [77–79]. PD subjects with the so-called “dopamine dysregulation syndrome” (i.e., subjects with abusive use of anti-PD medication) release large amounts of dopamine in the ventral striatum after levodopa administration [77]. It is known that the release of dopamine in the nucleus accumbens has rewarding effects [80], which would lead to the perpetuation of impulsive behaviours. Naturally, dopamine-related increases in the dopaminergic tone of the ventral striatum are expected to cause hyperactivation of ACC and OFC. In keeping with the PDFCD model, imaging studies in non-PD subjects with obsessive-compulsive disorder have shown decreased dopamine D2 binding—highly suggestive of increased dopamine levels—in the ventral striatum, and hyperactivation of OFC and ACC [81–84]. As the caudate nucleus modulates abnormal behaviours [5, 85], it could be tentatively argued that primarily compulsive behaviours (e.g., punding) might be more common in PDFCD stage I, and primarily reward-related ICDs (e.g., pathological gambling) might be more common in PDFCD stage IIa. In this context, ICDs in PD are associated with depression, anxiety, and obsessive-compulsive symptoms [86]. Clinical studies suggest that young PD subjects are at high risk of developing ICDs [76], perhaps in relation to age-dependent dopamine release dynamics [87]. Younger PD subjects release more dopamine, and at a faster rate, than older PD subjects.

Treatment-related visual hallucinations sometimes herald the development of dementia [6]. The PDFCD model suggests that, by the time the first hallucinations appear, all the dopamine-dependent frontostriatal loops are already dysfunctional (hypoactive) in the OFF state, whereas the dopaminergic projection to the frontal cortex is still preserved (PDFCD stage IIb; Figure 2). In this situation, frontal dopamine levels are expected to increase in response to dopaminergic treatment (ON state), possibly explaining why visual hallucinations are associated with relative frontal hypermetabolism [88]. Nonetheless, visual hallucinations in PD are also linked to hypometabolism in occipitotemporoparietal regions [89].

## 5. PDFCD Stage III: The Dopaminergic Projection to the Frontal Cortex

The damage to the direct dopaminergic projection to the frontal cortex signals the beginning of dementia [17, 18]. At this stage (PDFCD stage III; Figure 2), the patient begins to oscillate between dementia in the OFF state and psychosis in the ON state, a cycle that becomes more pronounced and fully established in more advanced PD, with the development of cortical Lewy body pathology. Other times, the patient remains apathetic during both OFF and ON periods.

In addition to striatal and frontal dopamine depletion, other neurotransmitters are also involved in PD dementia. Indeed, cortical cholinergic dysfunction can be even more severe in PD subjects with dementia than in patients with Alzheimer's disease [90, 91]. In advanced PD, cortical pathology—not only Lewy bodies but also neurofibrillary tangles and amyloid deposits—becomes a major contributing factor. Thus, in vivo PET studies have found comparable levels of cortical amyloid binding in patients with dementia with Lewy bodies and patients with Alzheimer's disease [92, 93].

## 6. Conclusions

The PDFCD model provides a systematic assessment of cognitive and behavioural symptoms, which may help clinicians optimize therapeutic strategies. It also provides a framework for hypothesis testing. For example, in prospective studies, apathy should not appear before ICDs in most patients. The model has a number of strengths and limitations. Among its strengths, it is simple and fits well clinical observations. For example, it explains why depression can be present during both OFF and ON periods, why depression and apathy are different disorders, and why some patients oscillate between apathy and depression or between dementia and psychosis. The combination of ON-period depression and impulsivity (PDFCD stage IIa) is relevant to explain why some PD subjects treated with subthalamic stimulation attempt suicide [94, 95]. Among its limitations, the model is in part *ad hoc* and does not contemplate region-specific assessments of the direct dopaminergic projection to the frontal cortex. In the early stages of PD, for example, cortical dopamine upregulation might only involve the DLPFC [32, 33]. There is evidence to suggest that the dopaminergic projection to the frontal cortex has limited capability for adaptation in response to dopaminergic treatment, particularly levodopa, due to its lack of dopamine transporter sites and dopamine D2 autoreceptors [38]. Still, treatment-related psychosis occurring in PDFCD stage III could be associated with relative dopaminergic hyperactivity in the frontal cortex. In other words, frontal cortex “overdose” may still be possible in later stages causing psychosis. Finally, the model assumes that dopamine-dependent frontostriatal functioning follows an inverted U-shaped dose-response curve. Whereas this type of dopamine response seems to operate in the frontal cortex [96], it might not necessarily occur in the striatum. Additional model refinements are certainly needed, including a better definition of stages IIa and IIb. To reach this goal, we need to develop better tools to reliably distinguish between depression and apathy [97] and reliably measure apathy and fatigue [98, 99].
